# Secretome data from *Trichoderma reesei* and *Aspergillus niger* cultivated in submerged and sequential fermentation methods

**DOI:** 10.1016/j.dib.2016.05.080

**Published:** 2016-06-08

**Authors:** Camila Florencio, Fernanda M. Cunha, Alberto C. Badino, Cristiane S. Farinas, Eduardo Ximenes, Michael R. Ladisch

**Affiliations:** a Laboratory of Renewable Resources Engineering, Department of Agricultural and Biological Engineering, Purdue University, 47907 West Lafayette, IN, USA; b Graduate Program of Biotechnology, Federal University of Sao Carlos, 13565-905 Sao Carlos, SP, Brazil; c Embrapa Instrumentation, 1452 XV de Novembro Street, 13560-970 Sao Carlos, SP, Brazil; d Graduate Program of Chemical Engineering, Federal University of Sao Carlos, 13565-905 Sao Carlos, SP, Brazil

**Keywords:** *Tricoderma reesei*, *Aspergillus Niger*, Enzyme Production, Secretome

## Abstract

The cultivation procedure and the fungal strain applied for enzyme production may influence levels and profile of the proteins produced. The proteomic analysis data presented here provide critical information to compare proteins secreted by *Trichoderma reesei* and *Aspergillus niger* when cultivated through submerged and sequential fermentation processes, using steam-explosion sugarcane bagasse as inducer for enzyme production. The proteins were organized according to the families described in CAZy database as cellulases, hemicellulases, proteases/peptidases, cell-wall-protein, lipases, others (catalase, esterase, etc.), glycoside hydrolases families, predicted and hypothetical proteins. Further detailed analysis of this data is provided in “Secretome analysis of *Trichoderma reesei* and *Aspergillus niger* cultivated by submerged and sequential fermentation process: enzyme production for sugarcane bagasse hydrolysis” C. Florencio, F.M. Cunha, A.C Badino, C.S. Farinas, E. Ximenes, M.R. Ladisch (2016) [1].

**Specifications Table**

**Table:** 

Subject area	Biochemistry
More specific subject area	Proteomic
Type of data	Table
How data was acquired	LC MS/MS analysis using Mascot Daemon version 2.4.0 (Matrix Science)
Data format	Analyzed
Experimental factors	Concentrated enzymatic cocktail from *A. niger* A12 and *T. reesei* Rut C30 produced by submerged fermentation and sequential fermentation
Experimental features	Peptides from enzyme cocktail of *A. niger* A12 and *T. reesei* Rut C30 were analyzed by LC-MS/MS
Data source location	Purdue University, West Lafayette, USA.
Data accessibility	Data is with this article

**Value of the data**

• This data set will be of value to the scientific community aiming to analyze the identified proteins secreted by *T. reesei* and *A. niger* under different cultivation methods.
• The data can be a useful tool to effectively select fungal strain and cultivation procedure for the production of proteins of interest.
• The data provided here identify key enzymes from *T. reesei* and *A. niger* for combined use to effectively degrade lignocellulose substrates, and therefore provide an opportunity to help researchers in the field to formulate enzyme cocktails in according to characteristics of lignocellulose biomass and enzyme activities found in the secretome.

## 1 Data

In Table 1, the proteins identified by proteomic analysis of enzymatic cocktails from *Trichoderma reesei* and *Aspergillus niger*, cultivated on pretreated sugarcane bagasse by either submerged or sequential fermentation processes, are presented according to the families classification from CAZy database.

The enzymatic hydrolysis of pretreated sugarcane bagasse was performed with combined extracts from *T. reesei* Rut C30 and *A. niger* A12, and the data of proteomic analysis of this combination of identified proteins is shown in Table 2. The indicated enzyme loadings were applied for steam-explosion sugarcane bagasse saccharification as described by Florencio et al. [1].

## 2 Experimental design, materials and methods

### 2.1 Fungal strains

The strains used for enzyme production were *T. reesei* Rut-C30 and *A. niger* wild type A12 obtained from Centre for Agricultural Bioscience International (CABI) culture collection (United Kingdom) and Embrapa Food Technology collection (Rio de Janeiro, Brazil), respectively. The conditions in which strains were maintained are described in Florencio et al. [1].

### 2.2 Cultivation conditions

Submerged and sequential fermentations carried out to obtain the enzymatic cocktails from *T. reesei* and *A. niger* are described in detail in Florencio et al. [1]. Briefly, the submerged fermentation was initiated with a 48 h pre-culture that contained a final conidia concentration of 10^7^ spores/mL in 100 mL of nutrient medium with 30 g/L of glucose, as described initially from Mandels and Stenberg [2] and adapted by Cunha et al. [3].

In the sequential fermentation, solid state fermentation was initiated using 5 g of dry sugarcane bagasse as solid substrate, and substrate moisture was adjusted through the addition of 12 mL of nutrient medium. The inoculum was added for a final concentration of 10^7^ spores/g of dry bagasse in the pre-culture, which was maintained under static conditions for 24 h. Then, the pre-culture step was continued as a submerged fermentation after the addition of 100 mL of nutrient medium enriched with 30 g/L of glucose per 5 g of dry bagasse. After 48 h for both submerged and sequential fermentation, a volume of pre-culture suspension corresponding to 10% (v/v) was transferred to 100 mL of culture medium for enzyme production, which was supplemented with 10 g/L of glucose and 1% (w/v) of steam-exploded non-washed sugarcane bagasse. All cultivation experiments were carried out in triplicate, and the averaged data presented with standard deviations.

## 3 Secretome analysis

### 3.1 Sample preparation

Sequence grade Lys–C/Trypsin (Promega) was used to enzymatically digest the samples. Acetone precipitation was performed prior to sample digestion. The protein samples were reduced with a 10 mM dithiothreitol (DTT)/25 mM ammonium bicarbonate solution at 37 °C for 1 h and alkylated at 37 °C also for 1 h using a solution of 97% acetonitrile (ACN), 2% iodoethanol, and 0.5% triethylphosphine (v/v). Samples were dried before adding Lys–C/trypsin to them in a 25:1 ratio of protease to protein. Digestions were carried out in a barocycler NEP2320 (PBI) at 50 °C and 20 kpsi for 2 h. The samples were cleaned over C18 columns (MicroSpin, Nest Group), dried and resuspended in 97% purified water/3% ACN/0.1% formic acid (FA). A volume of 1 µL was used for LC-MS/MS analysis.

### 3.2 LC-MS/MS analysis

A nanoLC system (1100 Series LC, Agilent Technologies, Santa Clara, CA) was used to separate the peptides for downstream MS analysis using a C18 reversed phase ZORBAX 300SB-C18 analytical column (0.75 μm×150 mm, 3.5 um) from Agilent. The column was directly connected to New Objective׳s emission tip coupled to the nano-electrospray ionization (ESI) source of the high resolution hybrid ion trap mass spectrometer LTQ-Orbitrap XL (Thermo Scientific). Elution was conducted using an ACN/0.1% FA (mobile phase B) linear gradient. The column was equilibrated initially for 5 min with 95% H_2_O /0.1% FA (mobile phase A) followed by the linear gradient of 5–40% B for 85 min at 0.3uL/min, then from 40–95% B for 12 min. Blank injections were performed in between experimental runs. The resulting eluents were analyzed by a data-dependent positive acquisition mode at full MS scan (30,000 resolution) where the eight most abundant molecular ions were selected and fragmented by collision induced dissociation (CID) using a normalized collision energy of 35% to acquire the data for the LTQ-Orbitrap XL.

### 3.3 Data analysis

Database search analyses were done using Mascot Daemon version 2.4.0 (Matrix Science) against an all fungal protein database from the NCBI database. Peptide and spectral count data were performed on the searches. For protein identification, at least two peptides detected were considered, and the false discovery rate (FDR) was set to 1%.

## Figures and Tables

**Table 1:** Major proteins identified in the secretome of *Trichoderma reesei* and *Aspergillus niger* cultivated under submerged (A) and sequential (B) fermentation methods.

**Gene ID**	**Enzyme**		**Family**	***T. reesei***		***A. niger***	
				**A**	**B**	**A**	**B**
21842121	Cellulases	Endoglucanase	GH12	x	x		
3757552		Endoglucanase A	GH12			x	x
145235569		Endo-beta-1,4-glucanase A	GH12				x
145228915		Endo-beta-1,4-glucanase A	GH12				x
2833231		Endoglucanase I	GH7	x	x		
121794		Endoglucanase II	GH5	x			
201066457		Endoglucanase IV (AA9)	GH61	x	x		
145235523		Glucan endo-1,3-beta-glucosidase eglC	–			x	x
320592482		Beta-glucanase	–	x			
403314396		Endoglucanase VI	GH61	x			
145229151		Endo-1,3(4)-beta-glucanase	GH16			x	x
202072834		Cellobiohydrolase I	GH7	x	x		
95115828		Cellobiohydrolase II	GH6	x	x		
74698499		1,4-beta-D-glucan cellobiohydrolase	GH7			x	x
201066459		Glucosidase	GH3	x			
126046487		β-glucosidase	GH3			x	x
145242946		β-glucosidase M 4	GH3				x
145255120		Glucan 1,3-beta-glucosidase A	GH5			x	x
400602153		Glucan 1,3-β-glucosidase	GH17	x			
257187		Alpha-glucosidase P2 subunit 5	GH31				x

317035725	Hemicellulases	Endo-arabinase	GH43				x
145234699		Alpha-L-arabinofuranosidase axhA	GH62			x	x
358375978		Arabinoxylan arabinofuranohydrolase	GH62			x	x
145233623		Endo-1,5-alpha-L-arabinosidase C	GH43			x	x
145250511		Alpha-N-arabinofuranosidase B	–			x	x
78101601		Anfaea-ferulic Acid Complex	–			x	
23821545		Feruloyl esterase B	–			x	
145246174		Feruloyl esterase C	–			x	x
48425840		Ferulic acid esterase	–				x
145247672		Feruloyl esterase B-1	–				x
145230716		Beta-galactosidase E	GH35			x	x
350630290		Alpha-galactosidase extracellular	–				x
74626383		Alpha-galactosidase B	–			x	x
317034650		Alpha-galactosidase D	–				x
307776646		Beta-mannanase	GH5	x	x		
358367813		Alpha-mannosidase	GH38			x	
145233855		Alpha-mannosidase	GH38			x	
572273984		Beta-mannosidase A	GH2	x			
572273001		Putative beta-mannosidase A	GH2	x	x		
317032967		Beta-mannosidase A	GH2			x	X
358369379		Beta-mannosidase (MndA)	GH2				x
145230794		Alpha-1,2-mannosidase 1B	GH47			x	x
145256261		Pectate lyase plyB	–			x	
572278177		Pectin lyase-like protein	–	x	x		
165906534		Endoxylanase	GH10	x	x		
11513450		Acetyl Xylan Esterase	–	x	x		
292495278		Endo-1,4-beta-xylanase C	GH10			x	x

549461	Hemicellulases	Endo-1,4-beta-xylanase 2	GH11	x	x		
145250044		Endo-1,4-beta-xylanase 5	GH11			x	x
157488002		Swollenin	CBM1	x	x		
9858848		Xylanase	GH11			x	
42716406		Xylanase	GH11	x	x		
13242071		Xylanase	GH11				x
26514830		Xylanase	GH11		x		
83638302		Xylanase	GH11				x
380293098		Xylanase II	GH11	x	x		
145242002		Alpha-xylosidase	GH31			x	x
145230215		Exo-1,4-beta-xylosidase xlnD	GH3			x	x
145243586		Xylosidase/arabinosidase	–			x	x

145228611	Proteases/ Peptidases	Aorsin	–				x
530795		Pepsinogen	–			x	x
589101183		Aminopeptidase	–	x			
145257498		Aminopeptidase 2	–			x	
145242728		Vaculolar aspartyl aminopeptidase Lap4	–			x	x
145583569		Aspartic endopeptidase	–	x			
145254317		Aspartic-type endopeptidase opsB	–				x
145248205		Aspartic-type endopeptidase opsB	–				x
145256471		Dipeptidyl peptidase III	–			x	
145249068		Tripeptidyl-peptidase sed2	–				x
629687989		Tripeptidyl peptidase precursor	–	x			
145246822		Extracellular serine carboxypeptidase	–			x	
1093596		Ser carboxypeptidase	–				x
145235505		Serine carboxypeptidase	–			x	x
317026828		Serine-type carboxypeptidase	–			x	x
134077081		Endoprotease Endo-Pro-A. niger	–			x	x
62002221		Subtilase protease	–		x		
115111226		Subtilisin-like protease	–	x	x		
589111601		Serine protease	–		x		
29421423		Extracellular serine protease	–	x			
124295071		SprT - serine protease	–	x	x		
464359		Subtilisin-like serine protease pepC	–			x	
589099267		Trypsin-like serine protease	–	x	x		
193735605		Vacuolar protease A	–		x		
387772861		Aspartic proteinase	–	x	x		

38256986	Cell-wall protein	Cell wall protein	–			x	
47028077		Cell-wall protein - CwpA	–				x
145252266		GPI anchored cell wall protein	GH64			x	x
589109601		Ceramidase family protein	–		x		
145255556		Alkaline nonlysosomal ceramidase	–			x	
387772865		Cerato-platanin	–	x	x		
270160616		Chitinase	GH18	x	x		
145232927		Endochitinase 1	GH18			x	x
1839391		Exochitinase	GH20	x			
145256696		Protein ecm33	–			x	x

145241592	Lipases	Lysophospholipase 1	–				x
145234164		Lysophospholipase 1	–				x
145231236		Phospholipase C PLC-C	–				x
109677003		Triacylglycerol lipase precursor	–				x
110431975		Triacyglycerol lipase B	–				x

589114715	Others	Amidase	–	x	x		
145239143		Aminotransferase, class V	–			x	
145241960		Alpha-amylase	–			x	
350631148		Alpha-amylase A	CBM20				x
145243632		Alpha-amylase a type-1/2	–			x	x
224027		Glucoamylase G1	GH15			x	x
145241784		N-acetylglucosaminidase	GH20			x	
113206519		Acetyl esterase	–		x		
589098125		Carbohydrate esterase	–	x	x		
358388255		Carbohydrate esterase family 15 protein	CBM15	x	x		
572279065		Carboxylesterase	–		x		
145233451		Cholinesterase	–			x	x
1705640		Catalase R	–				x
589115621		Catalase/peroxidase	–	x			
145228625		Catalase R	–			x	
119474019		Mycelial catalase Cat1	–			x	
404312830		Cellulose Induced Protein, CIP1	–	x	x		
589107171		Oxalate decarboxylase	–	x	x		
380482942		Oxalate decarboxylase family bicupin	–	x			
1169291		Aldehyde dehydrogenase	–			x	
572279542		Dihydrolipoyl dehydrogenase	–	x	x		
350631179		FAD/FMN-containing dehydrogenase	–			x	
589113573		Malate dehydrogenase	–	x			
19702487		Malate dehydrogenase			x		
145257405		Short-chain dehydrogenase	–			x	x
145230419		Glycosidase crf1	–				x
145256130		1,3-beta-glucanosyltransferase gel1	GH72			x	x
145240407		1,3-beta-glucanosyltransferase gel2	GH72			x	x
145241490		1,3-beta-glucanosyltransferase gel3	GH72				x
145234270		Glutaminase GtaA	–			x	x
145247260		Inulinase	GH32			x	x
145242650		Nucleoside diphosphate kinase	–			x	
589102565		Acid phosphatase-like protein	–	x	x		
130734		Phosphate-repressible acid phosphatase	–				x
145232002		Phosphatidylglycerol	–			x	x
145251519		Phosphoglycerate mutase family protein	–				x

572278887	Glycoside Hydrolases families	Glycoside Hydrolase (GH)	GH	x			
572275960		GH, partial	GH		x		
358381827		GH family 2 protein	GH2	x			
589104105		GH family 3	GH3		x		
358388254		GH family 5 protein	GH5	x			
589100793		GH family 10	GH10	x	x		
261825113		GH family 15 protein (glucoamylase)	GH15	x	x		
589113453		GH family 16	GH16	x			
358382969		GH family 16 protein	GH17	x			
589111611		GH family 17	GH17	x			
589113629		GH 18 protein (chitinase)	GH18	x	x		
317028062		GH, family 18	GH18			x	
589109851		GH family 28	GH28	x			
358380963		GH family 28 protein	GH28	x			
572273805		Family 31 GH	GH31	x	x		
589103027		GH family 38 protein	GH38		x		
358387943		GH family 43 protein	GH43	x			
589101105		GH family 47	GH47	x	x		
631371154		GH family 47 protein	GH47	x	x		
589100379		GH family 54 (lignin-degrading)	GH57	x	x		
589115645		GH family 55	GH55	x			
589114155		GH familiy 67	GH67		x		
358384989		GH family 71 protein	GH71	x			
589103161		GH family 71 protein	GH71	x			
589109155		GH family 71 protein	GH71	x	x		
589111135		GH family 72 (lignin-degrading)	GH72	x			
589108435		GH 74	GH74	x	x		
358380926		GH family 74 protein	GH74	x			
589098631		GH 92	GH92	x	x		
589100807		GH family 92	GH92	x			

255722211	Predicted proteins	Predicted protein	–				x
589105897		Predicted protein	–	x	x		
589101909		Predicted protein	–	x	x		
589110563		Predicted protein	GH16	x	x		
589113917		Predicted protein	–	x			
589109549		Predicted protein	GH67	x	x		
589108581		Pr Predicted protein	GH16	x			
403411875		Predicted protein	–	x			
589105505		Predicted protein	–	x			
589107107		Predicted protein	–	x	x		
589100041		Predicted protein	–	x	x		
589115849		Predicted protein	–	x			
589099057		Predicted protein	–	x			
589112857		Predicted protein	–	x			
589116001		Predicted protein	–	x			
589113291		Predicted protein	–		x		
589115927		Predicted protein	–		x		
154322591		Predicted protein	–		x		

358390109	Hypothetical proteins	Hypothetical protein TRIATDRAFT_129231	–	x			
358386311		Hypothetical protein TRIVIDRAFT_45439	–	x			
358390537		Hypothetical protein TRIATDRAFT_302472	–	x	x		
572280833		Hypothetical protein M419DRAFT_97005	–	x			
116199677		Conserved hypothetical protein	–	x			
589112113		Hypothetical protein TRIREDRAFT_66935	–	x	x		
358386247		Hypothetical protein TRIVIDRAFT_179276	–	x			
572280092		Hypothetical protein M419DRAFT_62371	–	x			
572273052		Hypothetical protein M419DRAFT_125562	–	x			
358380920		Hypothetical protein TRIVIDRAFT_118319	–	x			
572284103		Hypothetical protein M419DRAFT_94877	GH71	x	x		
589108875		Hypothetical protein TRIREDRAFT_122487	–	x			
380490319		Hypothetical protein CH063_07742	–	x			
358394718		Hypothetical protein TRIATDRAFT_300431	–	x			
345562011		Hypothetical protein AOL_s00173g184	CBM1		x		
440640361		Hypothetical protein GMDG_04666	–		x		
358381566		Hypothetical protein TRIVIDRAFT_49497	–		x		
358385331		Hypothetical protein TRIVIDRAFT_60255	–		x		
358388440		Hypothetical protein TRIVIDRAFT_141673	–		x		
358381654		Hypothetical protein TRIVIDRAFT_4609	–		x		
46127631		Hypothetical protein FG08193.1	–		x		
310800235		Hypothetical protein GLRG_10272	–		x		
598027367		Hypothetical protein AURDEDRAFT_162084	–		x		
646290693		Hypothetical protein BOTBODRAFT_162340	–		x		
598062595		Hypothetical protein SPAPADRAFT_57777	–		x		
350636308		Hypothetical protein ASPNIDRAFT_182100	GH43			x	
350629486		Hypothetical protein ASPNIDRAFT_47677	GH43			x	
350632025		Hypothetical protein ASPNIDRAFT_128537	–			x	x
145246196		Hypothetical protein ANI_1_1560104	–			x	
350635020		Hypothetical protein ASPNIDRAFT_197780	–			x	
568447829		Hypothetical protein AGABI2DRAFT_199975	GH3			x	
350631594		Hypothetical protein ASPNIDRAFT_53033	GH72			x	x
46122475		Hypothetical protein FG05615.1	–			x	
134082115		Hypothetical protein An15g00620	–			x	
350637823		Hypothetical protein ASPNIDRAFT_52061	GH75			x	x
145258972		Hypothetical protein ANI_1_2174184	–			x	x
145254751		Hypothetical protein ANI_1_1218164	–			x	x
145233749		Hypothetical protein ANI_1_1558024	–			x	x
350633910		Hypothetical protein ASPNIDRAFT_54865	–			x	x
350639816		Hypothetical protein ASPNIDRAFT_124700	–			x	
350638529		Hypothetical protein ASPNIDRAFT_119858	GH31				x
350638823		Hypothetical protein ASPNIDRAFT_205361	–				x
350636991		Hypothetical protein ASPNIDRAFT_56689	–				x
350633205		Hypothetical protein ASPNIDRAFT_55058	–				x
350629696		Hypothetical protein ASPNIDRAFT_126535	–				x
145243362		Hypothetical protein ANI_1_1704094	GH1				x
563290941		Hypothetical protein SBOR_8115	–				x
398407925		Hypothetical protein MYCGRDRAFT_30155	–				x
350636557		Hypothetical protein ASPNIDRAFT_53540	–				x

**Table 2:** Major proteins identified in the submerged (A) and sequential (B) fermentation enzymatic extracts from *Trichoderma reesei* + *Aspergillus niger*, which were used in the hydrolysis process of the pretreated sugarcane bagasse at a 1:5 ratio, respectively.

**Gene ID**	**Enzyme**		**Family**	***T. reesei*****+*****A. niger*****(1:5)**	
				**A**	**B**
21842121	Cellulases	Endoglucanase	GH12	x	x
3757552		Endoglucanase A	GH12	x	x
145235569		Endo-beta-1,4-glucanase A	GH12		x
145228915		Endo-beta-1,4-glucanase A	GH12		x
2833231		Endoglucanase I	GH7	x	x
121794		Endoglucanase II	GH5	x	
201066457		Endoglucanase IV (AA9)	GH61	x	x
145235523		Glucan endo-1,3-beta-glucosidase eglC	–	x	x
320592482		Beta-glucanase	–	x	
403314396		Endoglucanase VI (AA9)	GH61	x	
145229151		Endo-1,3(4)-beta-glucanase	GH16	x	x
202072834		Cellobiohydrolase I	GH7	x	x
95115828		Cellobiohydrolase II	GH6	x	x
74698499		1,4-beta-D-glucan cellobiohydrolase	GH7	x	x
201066459		Glucosidase	GH3	x	
126046487		β-glucosidase	GH3	x	x
145242946		β-glucosidase M 4	GH3		x
145255120		Glucan 1,3-beta-glucosidase A	GH5	x	x
400602153		Glucan 1,3-β-glucosidase	GH17	x	
257187		Alpha-glucosidase P2 subunit 5	GH31		x

317035725	Hemicellulases	Endo-arabinase	GH43		x
145234699		Alpha-L-arabinofuranosidase axhA	GH62	x	x
358375978		Arabinoxylan arabinofuranohydrolase	GH62	x	x
145233623		Endo-1,5-alpha-L-arabinosidase C	GH43	x	x
145250511		Alpha-N-arabinofuranosidase B	–	x	x
78101601		Anfaea-ferulic Acid Complex	–	x	
23821545		Feruloyl esterase B	–	x	
145246174		Feruloyl esterase C	–	x	x
48425840		Ferulic acid esterase	–		x
145247672		Feruloyl esterase B-1	–		x
145230716		Beta-galactosidase E	GH35	x	x
350630290		Alpha-galactosidase extracellular	–		x
74626383		Alpha-galactosidase B	–	x	x
317034650		Alpha-galactosidase D	–		x
307776646		Beta-mannanase	GH5	x	x
358367813		Alpha-mannosidase	GH38	x	
145233855		Alpha-mannosidase	GH38	x	
572273984		Beta-mannosidase A	GH2	x	
572273001		Putative beta-mannosidase A	GH2	x	x
317032967		Beta-mannosidase A	GH2	x	x
358369379		Beta-mannosidase (MndA)	GH2		x
145230794		Alpha-1,2-mannosidase 1B	GH47	x	x
145256261		Pectate lyase plyB	–		x
572278177		Pectin lyase-like protein	–	x	x
165906534		Endoxylanase	GH10	x	x
11513450		Acetyl Xylan Esterase	–	x	x
292495278		Endo-1,4-beta-xylanase C	GH10	x	x
549461		Endo-1,4-beta-xylanase 2	GH11	x	x
145250044		Endo-1,4-beta-xylanase 5	GH11	x	x

157488002	Hemicellulases	Swollenin	CBM1	x	x
9858848		Xylanase	GH11	x	
42716406		Xylanase	GH11	x	x
13242071		Xylanase	GH11		x
26514830		Xylanase	GH11		x
83638302		Xylanase	GH11		x
380293098		Xylanase II	GH11	x	x
145242002		Alpha-xylosidase	GH31	x	x
145230215		Exo-1,4-beta-xylosidase xlnD	GH3	x	x
145243586		Xylosidase/arabinosidase	–	x	x

572278887	Glycoside Hydrolases families	Glycoside Hydrolase (GH)	GH	x	
572275960		GH, partial	GH		x
358381827		GH family 2 protein	GH2	x	
589104105		GH family 3	GH3		x
358388254		GH family 5 protein	GH5	x	
589100793		GH family 10	GH10	x	x
261825113		GH family 15 protein (glucoamylase)	GH15	x	x
589113453		GH family 16	GH16	x	
358382969		GH family 16 protein	GH17	x	
589111611		GH family 17	GH17	x	
589113629		GH 18 protein (chitinase)	GH18	x	x
317028062		GH, family 18	GH18	x	
589109851		GH family 28	GH28	x	
358380963		GH family 28 protein	GH28	x	
572273805		Family 31 GH	GH31	x	x
589103027		GH family 38 protein	GH38		x
358387943		GH family 43 protein	GH43	x	
589101105		GH family 47	GH47	x	x
631371154		GH family 47 protein	GH47	x	x
589100379		GH family 54 (lignin-degrading)	GH57	x	x
589115645		GH family 55	GH55	x	
589114155		GH familiy 67	GH67		x
358384989		GH family 71 protein	GH71	x	
589103161		GH family 71 protein	GH71	x	

589109155	GH families	GH family 71 protein	GH71	x	x
589111135		GH family 72 (lignin-degrading)	GH72	x	
589108435		GH 74	GH74	x	x
358380926		GH family 74 protein	GH74	x	
589098631		GH 92	GH92	x	x
589100807		GH family 92	GH92	x	

255722211	Predicted proteins	Predicted protein	–		x
589105897		Predicted protein	–	x	x
589101909		Predicted protein	–	x	x
589110563		Predicted protein	GH16	x	x
589113917		Predicted protein	–	x	
589109549		Predicted protein	GH67	x	x
589108581		Predicted protein	GH16	x	
403411875		Predicted protein	–	x	
589105505		Predicted protein	–	x	
589107107		Predicted protein	–	x	x
589100041		Predicted protein	–	x	x
589115849		Predicted protein	–	x	
589099057		Predicted protein	–	x	
589112857		Predicted protein	–	x	
589116001		Predicted protein	–	x	
589113291		Predicted protein	–		x
589115927		Predicted protein	–		x
154322591		Predicted protein	–		x

358390109	Hypothetical proteins	Hypothetical protein TRIATDRAFT_129231	–	x	
358386311		Hypothetical protein TRIVIDRAFT_45439	–	x	
358390537		Hypothetical protein TRIATDRAFT_302472	–	x	x
572280833		Hypothetical protein M419DRAFT_97005	–	x	
116199677		Conserved hypothetical protein	–	x	
589112113		Hypothetical protein TRIREDRAFT_66935	–	x	x
358386247		Hypothetical protein TRIVIDRAFT_179276	–	x	
572280092		Hypothetical protein M419DRAFT_62371	–	x	
572273052		Hypothetical protein M419DRAFT_125562	–	x	
358380920		Hypothetical protein TRIVIDRAFT_118319	–	x	
572284103		Hypothetical protein M419DRAFT_94877	GH71	x	x
589108875		Hypothetical protein TRIREDRAFT_122487	–	x	
380490319		Hypothetical protein CH063_07742	–	x	
358394718		Hypothetical protein TRIATDRAFT_300431	–	x	
345562011		Hypothetical protein AOL_s00173g184	CBM1		x
440640361		Hypothetical protein GMDG_04666	–		x
358381566		Hypothetical protein TRIVIDRAFT_49497	–		x
358385331		Hypothetical protein TRIVIDRAFT_60255	–		x
358388440		Hypothetical protein TRIVIDRAFT_141673	–		x
358381654		Hypothetical protein TRIVIDRAFT_4609	–		x
46127631		Hypothetical protein FG08193.1	–		x
310800235		Hypothetical protein GLRG_10272	–		x
598027367		Hypothetical protein AURDEDRAFT_162084	–		x
646290693		Hypothetical protein BOTBODRAFT_162340	–		x
598062595		Hypothetical protein SPAPADRAFT_57777	–		x
350636308		Hypothetical protein ASPNIDRAFT_182100	GH43	x	
350629486		Hypothetical protein ASPNIDRAFT_47677	GH43	x	
350632025		Hypothetical protein ASPNIDRAFT_128537	–	x	x
145246196		Hypothetical protein ANI_1_1560104	–	x	
350635020		Hypothetical protein ASPNIDRAFT_197780	–	x	

568447829	Hypothetical proteins	Hypothetical protein AGABI2DRAFT_199975	GH3	x	
350631594		Hypothetical protein ASPNIDRAFT_53033	GH72	x	x
46122475		Hypothetical protein FG05615.1	–	x	
134082115		Hypothetical protein An15g00620	–	x	
350637823		Hypothetical protein ASPNIDRAFT_52061	GH75	x	x
145258972		Hypothetical protein ANI_1_2174184	–	x	x
145254751		Hypothetical protein ANI_1_1218164	–	x	x
145233749		Hypothetical protein ANI_1_1558024	–	x	x
350633910		Hypothetical protein ASPNIDRAFT_54865	–	x	x
350639816		Hypothetical protein ASPNIDRAFT_124700	–	x	
350638529		Hypothetical protein ASPNIDRAFT_119858	GH31		x
350638823		Hypothetical protein ASPNIDRAFT_205361	–		x
350636991		Hypothetical protein ASPNIDRAFT_56689	–		x
350633205		Hypothetical protein ASPNIDRAFT_55058	–		x
350629696		Hypothetical protein ASPNIDRAFT_126535	–		x
145243362		Hypothetical protein ANI_1_1704094	GH1		x
563290941		Hypothetical protein SBOR_8115	–		x
398407925		Hypothetical protein MYCGRDRAFT_30155	–		x
350636557		Hypothetical protein ASPNIDRAFT_53540	–		x
