# Supplementary material for: Secretome data from Trichoderma reesei and Aspergillus niger cultivated in submerged and sequential fermentation methods
Source: Data Brief. 2016 Jun 8;8:588–98. doi: 10.1016/j.dib.2016.05.080 (PMC4936598; doi:10.1016/j.dib.2016.05.080)
Supplement: Supplementary file 1 — Supplementary material [file mmc1.pdf]

PURDUE
UNIVERSITY

LABORATORY OF RENEWABLE RESOURCES ENGINEERING

Michael R. Ladisch, Ph.D.
Distinguished Professor and Director

May 18, 2016

Conflict of Interest

The authors declare they have no competing interests.

We confirm that the manuscript has been read and approved by all named authors and that there are no other persons who satisfied the criteria for authorship but are not listed. We further confirm that the order of authors listed in the manuscript has been approved by all of us.

We confirm that we have given due consideration to the protection of intellectual property associated with this work and that there are no impediments to publication, including the timing of publication, with respect to intellectual property. In so doing, we confirm that we have followed the regulations of our institutions concerning intellectual property.

We understand that the Corresponding Author is the sole contact for the Editorial process (including Editorial Manager and direct communications with the office). Dr. Michael Ladisch is responsible for communicating with the other authors about progress, submissions of revisions and final approval of proofs. We confirm that we have provided a current, correct e-mail address which is accessible by the Corresponding Author and which has been configured to accept e-mail from the journal.

Sincerely yours,

Michael Ladisch

Michael R. Ladisch
Distinguished Professor and Director

cc: Alberto C. Badino
Fernanda M. Cunha
Cristiane S. Farinas
Camila Florencio
Eduardo Ximenes

Potter Engineering Center, Room 216 ■ 500 Central Drive ■ West Lafayette, IN 47907-2022
(765) 494-7022 ■ Fax: (765) 494-7023
